# Improving Decision Speed, Accuracy and Group Cohesion through Early Information Gathering in House-Hunting Ants

**DOI:** 10.1371/journal.pone.0013059

**Published:** 2010-09-29

**Authors:** Nathalie Stroeymeyt, Martin Giurfa, Nigel R. Franks

**Affiliations:** 1 School of Biological Sciences, University of Bristol, Bristol, United Kingdom; 2 Centre de Recherches sur la Cognition Animale, Université de Toulouse, UPS, Toulouse, France; 3 Centre de Recherches sur la Cognition Animale, CNRS, Toulouse, France; University of Western Ontario, Canada

## Abstract

**Background:**

Successful collective decision-making depends on groups of animals being able to make accurate choices while maintaining group cohesion. However, increasing accuracy and/or cohesion usually decreases decision speed and vice-versa. Such trade-offs are widespread in animal decision-making and result in various decision-making strategies that emphasize either speed or accuracy, depending on the context. Speed-accuracy trade-offs have been the object of many theoretical investigations, but these studies did not consider the possible effects of previous experience and/or knowledge of individuals on such trade-offs. In this study, we investigated how previous knowledge of their environment may affect emigration speed, nest choice and colony cohesion in emigrations of the house-hunting ant *Temnothorax albipennis*, a collective decision-making process subject to a classical speed-accuracy trade-off.

**Methodology/Principal Findings:**

Colonies allowed to explore a high quality nest site for one week before they were forced to emigrate found that nest and accepted it faster than emigrating naïve colonies. This resulted in increased speed in single choice emigrations and higher colony cohesion in binary choice emigrations. Additionally, colonies allowed to explore both high and low quality nest sites for one week prior to emigration remained more cohesive, made more accurate decisions and emigrated faster than emigrating naïve colonies.

**Conclusions/Significance:**

These results show that colonies gather and store information about available nest sites while their nest is still intact, and later retrieve and use this information when they need to emigrate. This improves colony performance. Early gathering of information for later use is therefore an effective strategy allowing *T. albipennis* colonies to improve simultaneously all aspects of the decision-making process – i.e. speed, accuracy and cohesion – and partly circumvent the speed-accuracy trade-off classically observed during emigrations. These findings should be taken into account in future studies on speed-accuracy trade-offs.

## Introduction

Cohesive animal groups often have to make consensual decisions to prevent the group from splitting apart and to preserve the advantages of social life, even though collective decision outcomes may sometimes be sub-optimal for certain group members [1]. Group cohesion, speed and accuracy of decisions are fundamental aspects of consensus decision-making which may greatly affect the fitness of group members [2]. However, ensuring accuracy of decisions and maintaining group cohesion require time-consuming phases of both information gathering and pooling to accumulate evidence about the alternatives and ensure effective information flow within the group [1]. As a result, decision accuracy and group cohesion cannot usually be improved without sacrificing decision speed, and vice versa. Such trade-offs between speed and accuracy are commonplace in animal decision-making and information processing and occur at various scales of biological organization [1]–[5].

Speed-accuracy trade-offs in collective decision-making have recently received considerable attention and many experimental and theoretical studies have attempted to describe such trade-offs, identify their underlying causes and investigate optimal strategies to achieve a suitable compromise between speed and accuracy depending on the context [2]–[10]. All these studies shared the common assumption that information gathering should start simultaneously with the decision-making process, and have imposed this constraint experimentally by using naïve subjects. However, in natural conditions, individuals may already have some experience and/or knowledge of the alternatives before a choice has to be made; and this could considerably alter the dynamics and outcome of decisions. In this study, we experimentally investigated the effects of prior knowledge of the environment on speed, accuracy and group cohesion and their trade-offs in a collective decision-making process: nest emigration by the house-hunting ant *Temnothorax albipennis*.

*Temnothorax* ants dwell in fragile nests, such as hollow acorns, twigs or rock crevices, which are highly susceptible to disturbance [11]. When their current nest deteriorates, colonies select a new nest site using a distinct sequence of behaviors. After their nest has been destroyed, a minority of workers (‘scouts’) leave the old nest to look for suitable nest sites. When a scout has deemed a new site suitable, she starts recruiting other scouts to it by tandem running – a slow recruitment method whereby one leader teaches one follower the way from the old nest to the new site [12]. Each recruit then assesses the site independently [13] and may start recruiting as well. The population in the new site therefore gradually increases until it reaches a “quorum threshold” which triggers full commitment to that site. Scouts then switch from recruiting by tandem running to carrying nestmates and brood items from the old to the new nest, a fast recruitment method that allows quick relocation of the colony into its new home [2], [14]. Scouts have been shown to recruit more readily to higher quality than to lower quality nest sites (see e.g. [15]–[16]). This results in an amplificatory process leading to faster population growth in higher quality sites, in which the quorum threshold is reached earlier than in lower quality sites. As a result, all or most transport is usually directed towards the best available option [2], [14].

Several reasons justify the choice of nest relocation in *T. albipennis* as a model system to study the effects of previous knowledge of the environment on group cohesion and speed and accuracy of collective decisions. These parameters are indeed easy to measure in laboratory experiments (see e.g. [7], [17]). Additionally, when allowed to choose between two available nests of different qualities, colonies display a typical speed-accuracy trade-off and emphasize either speed or accuracy depending on the urgency of the situation [2], [4]–[5], [7], [9], [18]. Finally, Franks et al. [19] showed that *T. albipennis* colonies can gather information about available nest sites before emigrating, while their own nest is still intact – a phenomenon known as “reconnaissance”. In particular, colonies familiarized with low quality nest sites developed an aversion towards these sites and tended to avoid them later when they had to emigrate. However, the authors did not investigate how colony performance (i.e. speed, accuracy and cohesion) may be affected by such aversion; additionally, they were unable to detect a similar phenomenon for high-quality nest sites: colonies familiarized with high quality nest sites showed neither aversion nor attraction towards these sites in later emigrations [19].

In this study, we re-examined whether *T. albipennis* colonies can gather information about high quality nest sites prior to emigration by using a spatially complex exploration/emigration arena, contrasting with the simple square arena used in the study by Franks et al. [19]. More specifically, we investigated whether familiarization with high quality nest sites had an impact on colony performance in terms of emigration speed, nest choice accuracy and group cohesion. We found that familiarization with a single high quality nest site prior to emigration increased emigration speed in single choice emigrations (*experiment 1*) and led to biased nest choice and increased group cohesion in binary choice emigrations (*experiment 2*). We also found that familiarization with a high quality and a low quality nest sites prior to emigration led to increased group cohesion and improved both speed and accuracy of emigrations (*experiment 3*), in apparent contradiction with the classical implications of a speed-accuracy trade-off.

## Results

**Figure 1:**
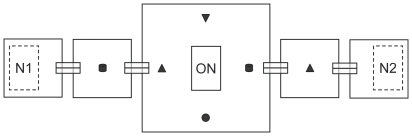
Experimental design. Top view of the exploration arena consisting of one large, central dish; two small, intermediate dishes; and two small, peripheral dishes. Adjacent dishes were connected by tunnels for the ants to walk through. Conspicuous landmarks (black shapes) were used to help ants orientate inside the arena. Colonies housed in their old nest (ON) were positioned in the middle of the central dish. One or two available new nest sites (N1 and N2) were positioned in the peripheral dishes either at the onset of exploration (familiar nests) or at the onset of emigration (unfamiliar nests). The position of new nest sites (right or left) was pseudo-randomized between colonies.

Colonies of *T. albipennis* housed in good nests were introduced to the middle of a symmetrical exploration arena consisting of several Petri dishes connected by tunnels (Figure 1). They were allowed to explore the design freely for one week until emigration was induced (see Materials and Methods). Emigrating colonies were presented with one good (experiment 1), two good (experiment 2), or one good and one mediocre (experiment 3) available new nest sites positioned in the peripheral dishes. For each colony, we monitored emigration dynamics and – when applicable – nest choice at the end of emigration under two treatments (see Materials and Methods for details on data recording). In the ‘naïve’ treatment, all new nest sites were introduced only at the onset of emigration; these nests, which are novel to all individuals at the time of emigration, are referred to as ‘unfamiliar’. In the ‘informed’ treatment, at least one new nest site was present in the arena during the whole exploration week; these nests, which may have been discovered and visited by individuals prior to emigration, are referred to as ‘familiar’. Table 1 summarizes the experimental protocols used in all experiments. In the ‘naïve’ treatment, all individuals in the colony were naïve regarding new nest sites at the time of emigration, whereas in the ‘informed’ treatment, some workers were informed and other were naïve depending on whether they had visited the familiar nest or not. However, for simplicity, the entire colony will hereafter be referred to as “naïve” or “informed” when presenting colony-level results.

**Table 1:** Experimental protocols.

					Available nest sites	
Experiment	*n*	*n*'	Old nest	Treatment	Exploration	Emigration
1	30	24	Good	Naïve	Ø	1 Good (U)
				Informed	1 Good	1 Good (F)
2	36	33	Good	Naïve	Ø	1 Good (U) +1 Good (U)
				Informed	1 Good	1 Good (F) +1 Good (U)
3	24	22	Good	Naïve	Ø	1 Good (U) +1 Mediocre (U)
				Informed	1 Good +1 Mediocre	1 Good (F) +1 Mediocre (F)

Total number of colonies used in the experiment (*n*) and in the final data analysis (*n*'); quality of the old nest; and number and quality of available nest sites during exploration and emigration for each experiment and each treatment (when applicable). For the emigration phase, it is indicated whether new nest sites are familiar (F) or unfamiliar (U).

### Experiment 1 – Prior Experience and Emigration Speed

In this experiment, colonies emigrated into one good new nest site positioned at one end of the arena; the opposite end, where there were no suitable nest site, was therefore a ‘dead end’.

**Figure 2:**
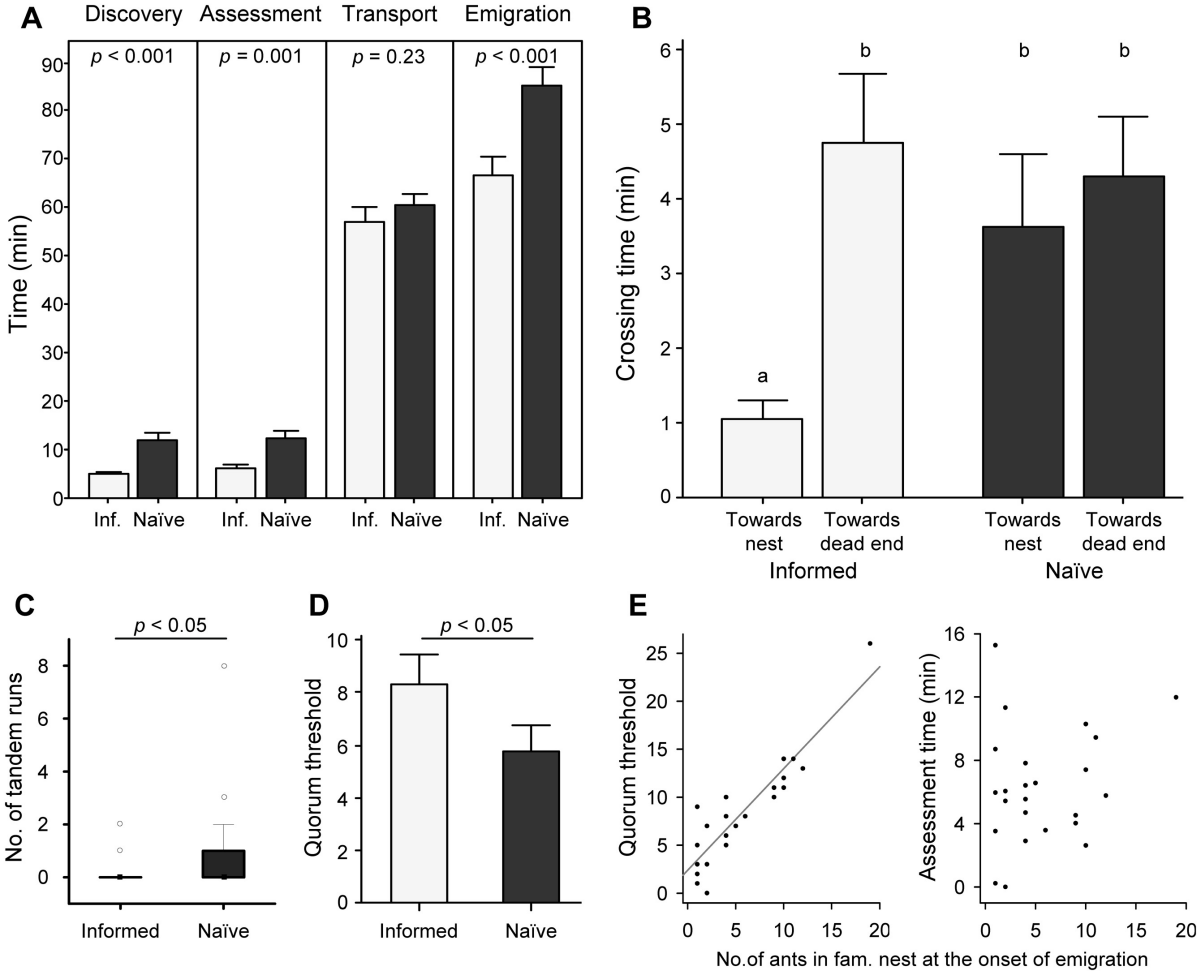
Prior experience and emigration speed (experiment 1). (**A–D**) Emigration data for informed (Inf., light grey, n = 24) and naïve (dark grey, n = 24) colonies emigrating to a single good nest site (experiment 1). Bars and whiskers represent the means and standard errors, respectively (A–B, D); full squares, rectangles, whiskers and open circles represent the median, interquartile range, 1.5 x interquartile range and outliers, respectively (C). (**A**) Discovery, assessment, transport and emigration times. The effect of treatment on each variable was tested using GLMM (no data transformation). (**B**) Crossing times of intermediate dishes leading either to the new nest site or to the dead-end. Same letters indicate no statistical differences, whereas different letters indicate significant statistical differences (*p*<0.05) in LSD post-hoc comparisons (GLMM, interaction treatment/direction: *p* = 0.059; no data transformation). (**C**) Number of successful forward tandem runs to the new nest site (Wilcoxon matched-pairs test). (**D**) Quorum thresholds used to switch to transport. The effect of treatment on quorum threshold was tested using GLMM (no data transformation). (**E**) Relationships between the number of ants in the familiar nest at the onset of emigration and, respectively, the quorum threshold (left) or the assessment time (right) for informed colonies (n = 24). Linear regression shows that these relationships are best described by the following equations: (i) Quorum Threshold  = 2.422+1.059 x No. of ants, *r^2^* = 0.82, *p*<0.001; and (ii) Assessment time  = 5.3+0.171 x No. of ants, *r^2^* = 0.048, *p* = 0.301.

Emigration was significantly faster for informed colonies, which were familiar with the new nest site, than for naïve colonies, which were unfamiliar with the new site (Figure 2A; GLMM, treatment: *p*<0.001). This was due to informed colonies discovering and assessing the new site faster than naïve colonies; by contrast, transport time did not differ between treatments (Figure 2A; GLMM, effect of treatment: discovery time, *p*<0.001; assessment time, *p* = 0.001; transport time: *p* = 0.23).

There were no differences in the crossing times of intermediate dishes (i.e. interval of time between the first entrance in the intermediate dish and the first entrance in the adjacent peripheral dish) leading to the unfamiliar nest and to the dead end for naïve colonies (random exploration; GLMM, LSD post-hoc comparison: *p* = 0.53; Figure 2B). Additionally, crossing times of intermediate dishes leading to the dead end for informed colonies were similar to the crossing times observed in naïve colonies (GLMM, LSD post-hoc comparisons, dead-end (informed)/dead-end (naïve): *p* = 0.66; dead-end (informed)/unfamiliar nest (naïve): *p* = 0.29; Figure 2B). By contrast, crossing times were significantly shorter for intermediate dishes leading to the familiar nest in informed colonies (GLMM, LSD post-hoc comparisons, *p*<0.05 in all comparisons; Figure 2B). Faster discovery of the new site in the ‘informed’ treatment was therefore not due to more effective exploration in all directions; rather, specific information on the position of the familiar nest allowed some individuals to head more quickly towards the nest.

During the assessment period, there were fewer forward tandem runs towards familiar nests (informed) than towards unfamiliar nests (naïve; Figure 2C; Wilcoxon matched-paired test, Z = −2.2, *p* = 0.028). Nevertheless, the quorum thresholds used in the ‘informed’ treatment were higher than those used in the ‘naïve’ treatment (Figure 2D; GLMM, effect of treatment: *p* = 0.014). This apparent contradiction may be explained because there were usually several workers inside the familiar nest at the onset of emigration. There was indeed a strong correlation between quorum threshold and number of workers in the familiar nest at the onset of emigration for informed colonies (Figure 2E; Pearson correlation coefficient r = 0.906; *p*<0.001). By contrast, we could not detect any correlation between assessment time and number of workers in the familiar nest at the onset of emigration (Figure 2E; Pearson correlation coefficient r = 0.220; *p* = 0.3). The faster assessment observed in the ‘informed’ treatment cannot therefore be solely explained by the presence of workers in the familiar nest at the onset of emigration already constituting a quorum threshold.

### Experiment 2 – Prior Experience, Nest Choice and Cohesiveness

In this experiment, colonies were offered a choice between two identical good new nest sites positioned at either ends of the arena. Informed colonies were familiar with one of these two nests, whereas naïve colonies were unfamiliar with both nests.

**Figure 3:**
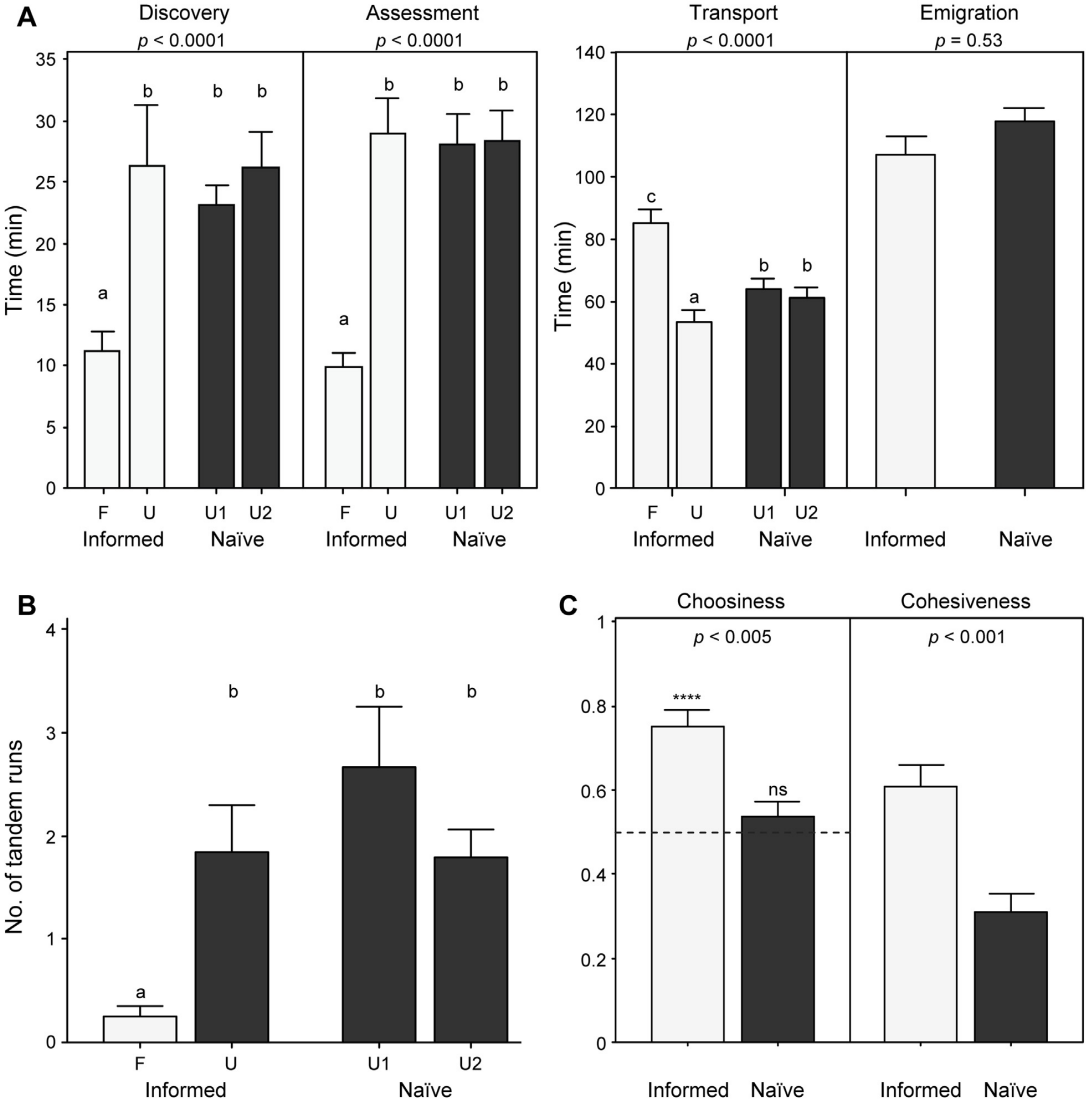
Prior experience, nest choice and cohesiveness (experiment 2). Emigration data for informed (light grey, n = 33) and naïve (dark grey, n = 33) colonies emigrating to one familiar (F) and one unfamiliar (U) good nests or to two unfamiliar good nests (U1 and U2), respectively (experiment 2). Bars and whiskers represent the means and standard errors, respectively. (**A**) Discovery, assessment, transport and emigration times. *P-*values are given for the effect of nest site (familiar/unfamiliar) on discovery, assessment and transport times, and the effect of treatment (naïve/informed) on emigration time (GLMM; discovery time was log-transformed). Same letters indicate no statistical differences, whereas different letters indicate significant statistical differences (*p*<0.05) in LSD post-hoc comparisons. (**B**) Number of successful forward tandem runs towards new nest sites. Same letters indicate no statistical differences, whereas different letters indicate significant statistical differences (*p*<0.05) in LSD post-hoc comparisons (GLMM; effect of nest: *p*<0.005; no data transformation). (**C**) Choosiness and Cohesiveness indexes. Choosiness was calculated as the proportion of items in the familiar nest (informed colonies) or in unfamiliar nest 1 (naïve colonies). *P*-values are given for the effect of treatment on both variables (GLMM; no data transformation). The broken line over choosiness – set at 0.5 – represents expectations under the hypothesis of random choice between both nests (*****: *p*<0.001 in one-sample Wilcoxon test for non-normal data; ns: non-significant in one-sample t-test for normal data).

Discovery and assessment were significantly faster for familiar than for unfamiliar nests (Figure 3A). Additionally, there were significantly fewer forward tandem runs to the familiar than to the unfamiliar nests (Figure 3B). Overall emigration time, however, did not differ between informed and naïve colonies (Figure 3A). This was due to the high initial splitting rate of colonies (29 out of 33 informed colonies and all naïve colonies (n = 33) split – reunion of split colonies occasionally occurred within 24 hours), which resulted in uneven transport effort between both nests in informed colonies and even transport effort in naïve colonies (Figure 3A). The resulting differences in transport time cancelled out the effect of faster discovery and assessment for the familiar nests.

Overall, naïve colonies chose randomly between the two unfamiliar nests (one-sample t-test: t = 1.134, df  = 32, *p* = 0.265) whereas informed colonies showed a significant preference for the familiar nest (one-sample Wilcoxon test: WS  = 521, *p*<0.001); informed colonies were significantly more choosy than naïve colonies (Figure 3C; GLMM, effect of treatment on choosiness: *p* = 0.003). Additionally, informed colonies were significantly more cohesive than naïve colonies (Figure 3C; GLMM, effect of treatment on cohesiveness: *p* = 0.001).

### Experiment 3 – Prior Experience and Speed-Accuracy Trade-Off

In this experiment, colonies were offered a choice between one good and one mediocre new nest sites positioned at either end of the arena. Informed colonies were familiar with both nests, whereas naïve colonies were unfamiliar with both nests.

**Figure 4:**
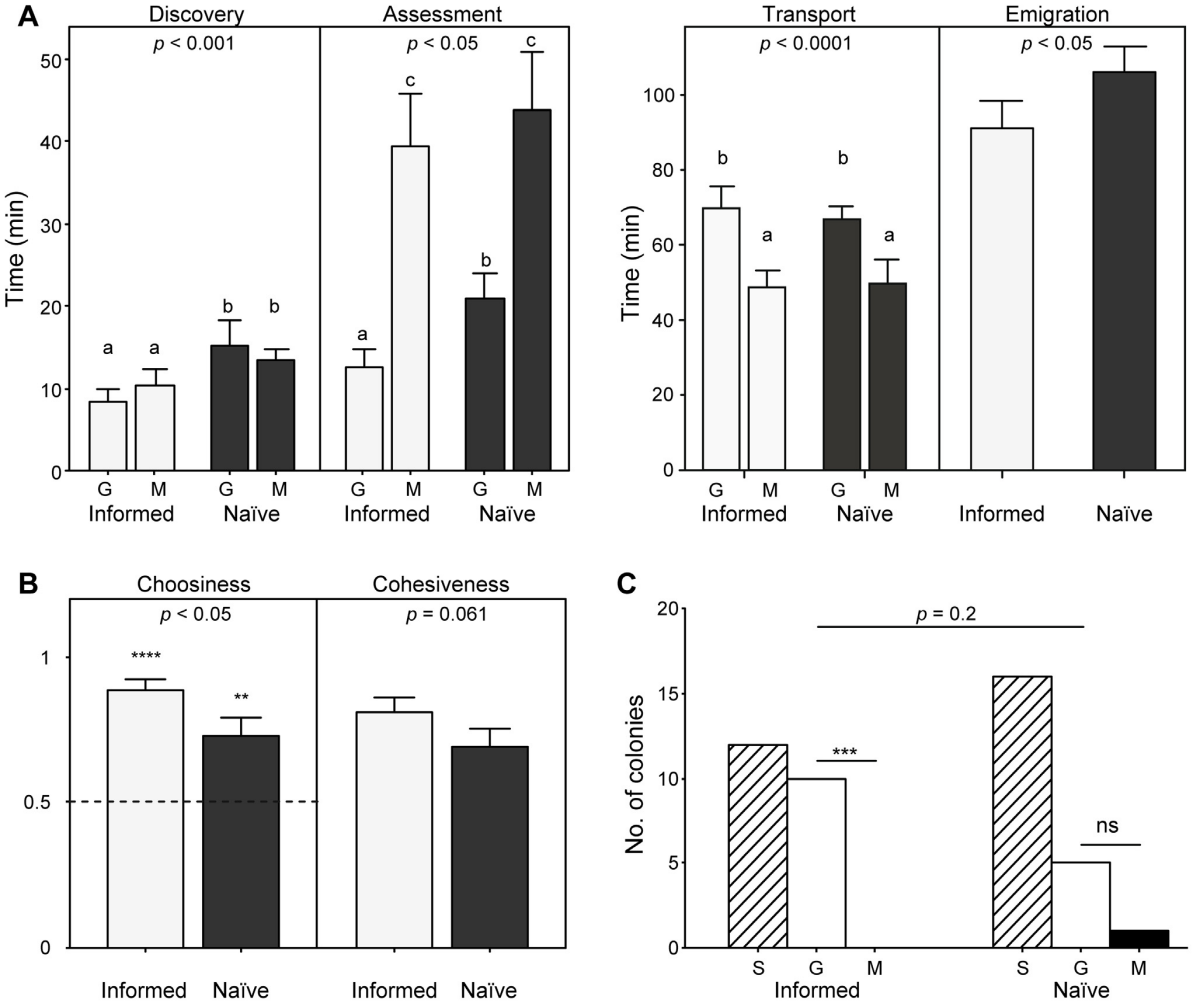
Prior experience and speed-accuracy trade-off (experiment 3), emigration. (**A–B**) Emigration data for informed (light grey, n = 22) and naïve (dark grey, n = 22) colonies emigrating to one good (G) and one mediocre (M) nest sites (experiment 3). Bars and whiskers represent the means and standard errors, respectively. (**A**) Discovery, assessment, transport and emigration times (discovery, assessment and transport are considered for each site whereas emigration time is considered for each colony). *P*-values are given for the effects of: (i) interaction between nest quality and treatment on assessment time; (ii) nest quality on transport time; and (iii) treatment on discovery and emigration times (GLMM; discovery and assessment times were log- and power-transformed, respectively). Same letters indicate no statistical differences, whereas different letters indicate significant statistical differences (*p*<0.05) in LSD post-hoc comparisons. (**B**) Choosiness and Cohesiveness indexes. Choosiness was calculated as the proportion of items in the good nest. Cohesiveness was calculated as described in the Materials and Methods section. *P*-values are given for the effect of treatment on both variables (GLMM; choosiness was power-transformed). The broken line over choosiness – set at 0.5 – represents expectations under the hypothesis of random choice between both nests (****: *p*<0.001; **: *p*<0.01 in one-sample Wilcoxon tests). (**C**) Number of colonies splitting (S, hashed bars) or choosing the good (G, white bars) or mediocre nest (M, black bars) at the end of emigration. Nest choice patterns were compared between treatments using Fisher-Freeman-Halton's exact test and nest preference was tested within each treatment using exact binomial tests (ns: non-significant; ****: *p*<0.001).

Emigration was significantly faster for informed colonies than for naïve colonies (Figure 4A; GLMM, effect of treatment: *p* = 0.019).

Familiar nests (both good and mediocre) were discovered earlier than unfamiliar nests (Figure 4A; GLMM, effect of treatment: *p*<0.001). Assessment time was longer for mediocre nests than for good nests; additionally, assessment of familiar good nests was faster than assessment of unfamiliar good nests (Figure 4A). Because transport started earlier for good than for mediocre nests, but ended simultaneously when all brood items had been carried away from the old nest, transport time was significantly longer for good than for mediocre nests (Figure 4A).

At the end of emigration, nest choice pattern did not differ between informed and naïve colonies (Figure 4C; Fisher-Freeman-Halton's test: *p* = 0.2). However, there was a significant preference of colonies for good over mediocre nests in the ‘informed’ treatment (binomial test: *p* = 0.002) but not in the ‘naïve’ treatment (binomial test: *p* = 0.22). Additionally, taking into account data on split colonies showed that both informed and naïve colonies preferred good nests (Figure 4B; one-sample Wilcoxon tests, test: *p*<0.001; naïve: *p* = 0.008), but informed colonies did so significantly more than naïve colonies (Figure 4B; GLMM, effect of treatment: *p* = 0.017). In other words, informed colonies were better at selecting the better option than naïve colonies. Informed colonies were also marginally more cohesive than naïve colonies (Figure 4B; GLMM, effect of treatment: *p* = 0.061).

During emigrations, informed colonies which had familiarized themselves with both the good and the mediocre nests were therefore (i) faster and (ii) more accurate than naïve colonies which were unfamiliar with both nests.

**Figure 5:**
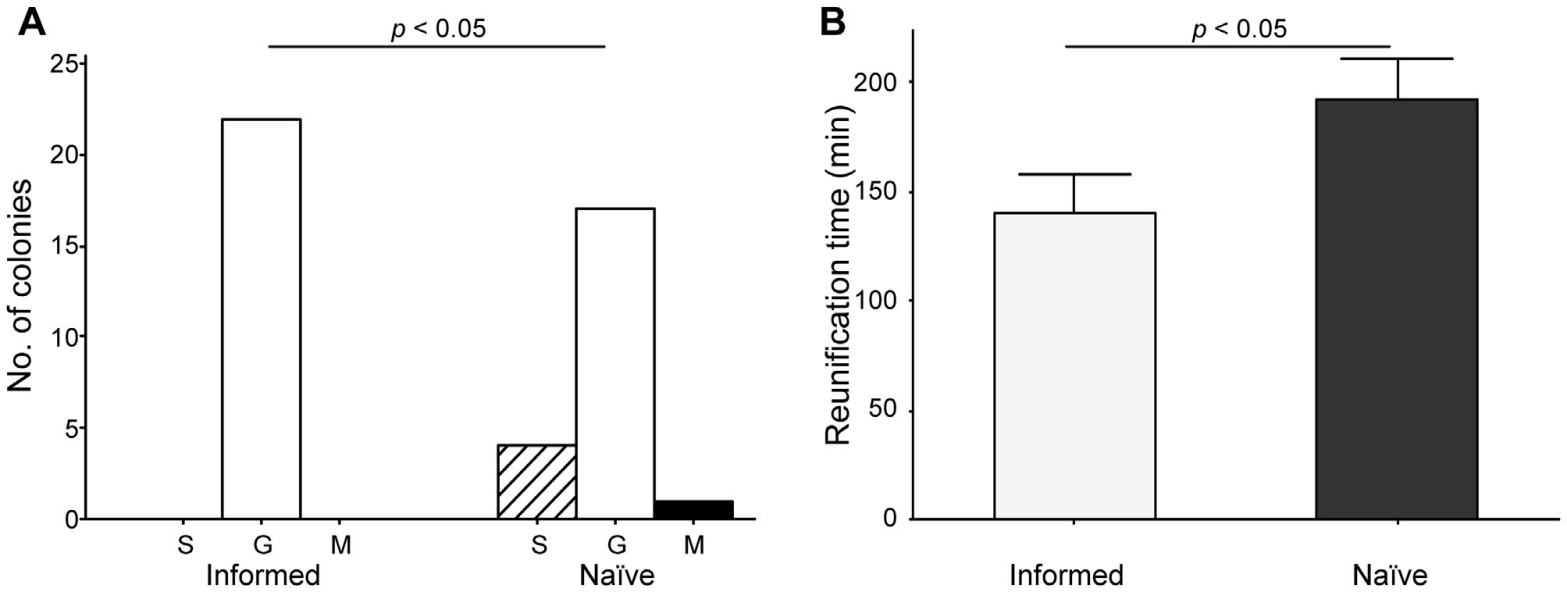
Prior experience and speed-accuracy trade-off (experiment 3), final state. (**A**) Number of colonies split (S, hashed bars) or having chosen the good (G, white bars) or mediocre nest (M, black bars) 24 hours after emigration onset. Nest choice patterns were compared between treatments using Fisher-Freeman-Halton's exact test and nest preference was tested within each treatment using exact binomial tests (ns: non-significant; ****: *p*<0.001). (**B**) Reunification time for informed (light grey, n = 18) and naïve (dark grey, n = 18) colonies. Bars and whiskers represent the means and standard errors, respectively. The effect of treatment on reunification time was tested using GLMM (no data transformation).

After 24 hours, all informed colonies (n = 22) had chosen the good nest whereas only 17 out of 22 naïve colonies had chosen the good nest (Figure 5A; Fisher-Freeman-Halton's exact test: *p* = 0.049). Additionally, reunification time was significantly shorter for informed than for naïve colonies (Figure 5B; GLMM, effect of treatment: *p* = 0.016). Informed colonies were therefore able to reunite faster and more successfully than naïve colonies.

## Discussion

Our results show that colonies of *T. albipennis* gather information about the location of available good nest sites prior to emigration, while their own nest is still intact, and can later retrieve and use that information when they have to emigrate. In all experiments, emigrating colonies indeed discovered familiar good nest sites faster than sites they had never encountered before. This was due to directed, i.e. non-random, exploration towards familiar sites.

Additionally, assessment times (time interval between the first discovery of a nest and full commitment to that nest) were shorter, and workers led fewer tandem runs, for familiar than for otherwise identical unfamiliar good nest sites. This indicates that at the onset of emigrations, colonies already had information on the suitability of high quality nest sites they had familiarized themselves with.

These results confirm that reconnaissance and prior experience affect nest emigration in *T. albipennis*, as previously shown in several studies [19]–[20]. However, the observation that colonies learnt the location and suitability of good nest sites contrasts with a previous study by Franks et al. [19], who were unable to detect an effect of familiarization with good nest sites on colony performance. This is because in that study, the exploration arena used was small and geometrically simple (22×22 cm dish). As a result, both familiar and unfamiliar nests were easy to find and there was little benefit in previous exploration of the familiar nest. By contrast, the exploration arena used in the present study was larger and geometrically more complex (Figure 1; one 22×22 cm dish was connected at opposite ends to two intermediate and two peripheral 10×10 cm dishes via a series of 4 cm long tunnels). This made new nest sites more difficult to find as they were separated from the old nest by two narrow tunnels. The advantages derived from previous knowledge of the location of the familiar nest should therefore be much higher, explaining the difference between this and previous studies.

Gathering information on the location and suitability of available high quality sites prior to emigration had a strong impact on colony performance during emigrations, i.e. on group cohesion, emigration speed and decision accuracy. When only one good new nest was available (experiment 1), colonies which had previously been in contact with that nest emigrated faster than naïve colonies. When there was a choice between two identical good new nests (experiment 2), colonies which had previously been in contact with one of these two nests showed a clear preference for that nest and remained more cohesive than naïve colonies, which chose randomly between both nests. Experiments 1 and 2 therefore showed that familiarization with a high quality nest could improve emigration speed and group cohesion independently, but did not reveal how both parameters could be affected simultaneously. In experiment 3, we allowed to colonies to choose between a good and a mediocre nests. Colonies which were familiar with both nests were faster, better at selecting the good nest and were marginally more cohesive than naïve colonies, both during the emigration process and at long term. In these specific experimental conditions (arguably more realistic than those in experiments 1 and 2, because colonies in their natural environment should encounter several suitable nesting cavities of different qualities) gathering information prior to emigration therefore allowed to improve simultaneously speed, accuracy and group cohesion.

These results are quite striking, as speed and accuracy of *T. albipennis* emigrations have repeatedly been shown to be subject to a trade-off, i.e. nest choice accuracy (and group cohesion) cannot be improved without having to spend more time in the decision-making process. The existence of such constraint on *T. albipennis* emigrations has received much support, both experimentally and theoretically [2]–[7], [9], [18] and is in keeping with the presence of similar speed-accuracy trade-offs in emigrations by other house-hunting social insects (ants: [10]; bees: [8]). Speed-accuracy trade-offs are widespread in living organisms and affect all levels of biological organization [3], from information processing in cells and nervous systems [4], [21]–[24] to decision-making in individuals [24]–[28] and groups of individuals [1], [3]; this is because gathering information in order to reach a decision is a time-consuming, noisy process, and increasing the accuracy of decision requires to spend more time accumulating evidence. Because the inherent property of a speed-accuracy trade-off is that one parameter cannot be improved without sacrificing the other, animals need to find a good compromise between both parameters depending on the costs incurred by inaccurate choices and/or slow decisions. Strategies for decision-making may therefore vary between individuals (or groups of individuals) [25]–[27], [29], but may also vary within a single individual (or group of individuals), depending on the context, to meet the requirements of specific situations by emphasizing either speed or accuracy [3], [7], [27]–[28]. Here, however, *T. albipennis* colonies appear to apply a strategy (i.e. gathering information long before the start of the decision-making process) which allows them to improve both parameters simultaneously.

The gap in time between information collection and exploitation is the key to improving both speed and accuracy of emigrations in *T. albipennis*: colonies indeed pay most of the time costs of discovering and assessing nests in advance – while their nest is still intact – which allows decisions to be both swift and accurate later when they need to emigrate. Time gaps between gathering and exploiting information have already been described in solitary species, such as prospecting birds [30] and some parasitoid wasps [31]. In both cases, this phenomenon derives mainly from important time constraints on information availability. Prospecting birds, for example, inspect various breeding patches and assess the reproductive success of conspecifics using social cues at the end of the breeding season. This influences their settlement choice in the next year: most prospecting birds indeed choose to settle closer to higher quality patches. The reason for such early prospecting is that the best cues for predicting breeding patch quality are social cues, which are not present at the time of the settlement but can only be monitored at the end of the previous breeding season [30], [32]–[34]. Parasitoid wasps are also subjected to time constraints. *Hyposoter horticola*, for example, needs to oviposit into its host's eggs at a very specific developmental stage of short duration. Similarly, parasitoid wasps *Argochrysis armilla* need to enter nests of digger wasps *Ammophila* sp. in the brief period between their host bringing a caterpillar back to its nest and sealing it. Because the period during which oviposition is possible is very short and therefore precludes search at that stage, these parasitoid wasps need to learn the location of their hosts' eggs or nests in advance and monitor them regularly [31]. Such time constraints on information availability may explain why solitary species may gather information well before they need to use it, in spite of the high potential costs incurred by such early search. Any time and energy spent on searching is indeed diverted from present reproduction and maintenance, which may have substantial fitness costs [30], [35]–[36]. However, these costs should be compensated for because early gathering of information is likely to greatly enhance future reproduction [30]–[31].

This situation contrasts with that of *T. albipennis* ants, which are not subject to time constraints on information availability. In natural conditions, suitable nest sites are indeed permanently accessible to workers, and nest site quality is best predicted by its present physical properties such as light level, headroom and entrance width [17]. Additionally, naïve colonies have been repeatedly shown to be able to assess, choose and relocate effectively to new nest sites during an emigration, even if they have never encountered these sites before [15]–[17], [37]–[42]. There is therefore no absolute necessity for colonies to gather information about nest sites prior to emigration. Even more strikingly, contrary to the solitary species mentioned above, which are preparing for a certain event (future reproduction), *T. albipennis* colonies gather information for later emigrations, which are uncertain events: an emigration may indeed not occur at all if the nest remains intact throughout the season [11]. Why, therefore, pay the costs of early information gathering [43] if the benefits associated with it are limited and may never be obtained? One answer is that even though the need for emigration may be unpredictable, they are probably quite frequent in *Temnothorax* species, especially those living in temporary nests such as hollow acorns and twigs. Additionally, the social organization of ants colonies, based on division on labor [44]–[48], reduces considerably the costs associated with information gathering while the nest is still intact. Exploration of available nest sites may indeed be time consuming and require energy, but it can be carried out with little extra cost by the same individuals that go out of the nest on a daily basis to explore and perform indispensable tasks such as foraging and patrolling. Visiting and assessing nest sites by patrollers and/or foragers while the nest is still intact should therefore be less costly – in terms of both time and energy – than during emergency emigrations, where it involves considerable efforts by many individuals (up to a 40% of a colony's total workforce [6]). Additionally, the potential consequences of time delays differ drastically between the two situations: while the nest is intact, most of the colony (and especially the queen) is safe inside the nest and can be effectively defended by a few individuals positioned at the entrance [17]. By contrast, during emergency emigrations, the entire colony is exposed so any time delays associated with information gathering may increase risk and incur higher costs to the vulnerable colony [48]. Gathering information while the nest is still intact should therefore greatly increase colony performance during later emigrations by simultaneously improving emigration speed, decision accuracy and group cohesion at relatively low costs and risks.

Our results imply that information about available nest sites is continually gathered by exploring individuals while the nest is still intact, then retrieved and shared among scouts during emigrations so as to affect the whole colony's performance. Information about suitable nest sites should therefore be available at any time and relatively easy to transfer among colony members. A previous study by Franks et al. [19] suggested that both chemical marking and visual cues may be involved in storing and retrieving information about low quality nest sites. Similarly, chemical marking and/or memory by informed individuals could play an important role in retrieving information about high quality nest sites. Other social cues (such as interactions among workers in new nest sites) could also be partly responsible for the faster assessment of high quality nest sites we observed in our experiments: in most cases a few workers were stationed inside good familiar nest sites at the onset of emigrations; these workers could help reach the quorum threshold faster than if the site was empty. However, although the number of workers present in the nest at the onset of emigration was strongly correlated with the quorum threshold, we did not find any evidence that it had an influence on assessment time. Further investigations will be necessary to determine what form(s) of information is (are) stored and the relative roles of informed versus naïve individuals in emigrations to high-quality, familiar nest sites [49].

Emigrations by *Temnothorax* ants represent one of the main sources of inspiration for theoretical models on speed-accuracy trade-offs in collective decision-making, aiming at identifying the sources of such trade-offs and possible optimal strategies to compromise between speed and accuracy [2]–[5], [9], [18]. However, all these models consider that colonies are totally naïve at the beginning of emigrations. The present study shows that in natural conditions, this may not be the case, as colonies are able to store information about available nest sites of different quality prior to emigration, then retrieve and use that information during emigrations, which in some cases allows improving both speed and accuracy of the decision-making process. We believe that previous knowledge of the environment should be taken into account in theoretical as well as experimental work on speed-accuracy trade-offs in collective decision-making, and hope the present work will stimulate new studies considering this issue.

## Materials and Methods

Ninety colonies of *T. albipennis* were collected in Dorset, UK, in spring and summer 2008 and 2009 and brought to Bristol, UK, where they were kept in the laboratory as described in [17].

### Nests and exploration arenas

Colonies were housed in artificial nests consisting of a cardboard perimeter sandwiched between two glass slides (50×76 mm) with an internal cavity of 35×50 mm and an entrance of 2×8 mm. All experimental nests had a paper floor between the cardboard perimeter and the bottom slide. *T. albipennis* colonies have been shown to consistently prefer nests with a dark interior over bright nests [17]. Accordingly, we designed two types of nests of different quality: ‘good nests’ were covered with a top sheet of cardboard so their nest cavity was dark, whereas ‘mediocre nests’ had no such cover and were therefore bright. At the beginning of all experiments, colonies were housed in good nests.

Experiments were performed under natural sunlight in exploration arenas consisting of large and small Petri dishes (respectively 22×22×2.2 cm and 10×10×1.7 cm) interconnected by tunnels (Figure 1). Each tunnel was made of two spectrometry cuvets positioned side by side and whose base was cut off to allow ants to walk through them. Tunnels fitted tightly through the walls of adjacent dishes, and any gaps between tunnels and dish walls were filled with silicone. Petri dishes were covered with lids and their walls were coated with Fluon to prevent ants from escaping.

### General experimental protocol

Colonies housed in their old nest were positioned in the middle of the central dish (Figure 1) and allowed freely to explore the arena. Six conspicuous landmarks painted with black powder paint (two cylinders of 26 mm diameter by 14 mm height; two cones and one inverted cone of 25 mm base and 12 mm height; and one truncated sphere of 18 mm diameter; disposed as shown in Figure 1) were interspersed in the arena to help the ants orientate. Colonies were provided with diluted honey, drosophila and water placed on top of their nest so that food position would not influence exploration pattern.

After one week's exploration, colonies were induced to emigrate by removing the top glass and cardboard perimeter of their old nest. At the onset of emigration, food trays and water tubes were removed from the arena and all workers observed in the intermediate or peripheral dishes were gently taken with soft forceps and released in the central arena. Emigrating colonies were presented with one (experiment 1) or two (experiments 2 and 3) available new nest sites positioned in the peripheral dishes of the arena (Figure 1). New nest sites were introduced in the arena either at the onset of exploration, so that they could be discovered and visited by individuals for one week before emigration (‘familiar’ nests), or at the onset of emigration, so that they were novel to all individuals in the colony at the time of emigration (‘unfamiliar’ nests).

Three different experiments were run (Table 1). In experiment 1, 30 colonies were allowed to emigrate into one good new site under two treatments: in the ‘informed’ treatment, colonies had familiarized themselves with new site before emigration, whereas in the ‘naïve’ treatment colonies were unfamiliar with that nest. In experiment 2, 36 colonies were allowed to choose between two identical good new sites under two treatments: in the ‘informed’ treatment, colonies had familiarized themselves with one of the two new sites, whereas in the ‘naïve’ treatment colonies were unfamiliar with both nests. In experiment 3, 24 colonies were allowed to choose between one good and one mediocre new sites under two treatments: in the ‘informed’ treatment colonies had familiarized themselves with both nests whereas in the ‘naïve’ treatment colonies were unfamiliar with both nests. As some colonies consistently displayed little activity during the exploration period, we excluded from later analyses those colonies in which no workers were observed in the peripheral dishes or (if applicable) in the familiar nest(s) at the onset of emigration. Additionally, some colonies emigrated into the new sites during the exploration period; those colonies were also excluded from the final analysis. The number of colonies used in the later analyses was therefore 24 in experiment 1; 33 in experiment 2; and 22 in experiment 3 (Table 1).

In all experiments, colonies were tested each under both treatments. Half of the colonies received the ‘naïve’ treatment first, whereas the other half received the ‘informed’ treatment first. All experiments consisted of successive replicates where 6 to 10 colonies explored and emigrated simultaneously in a single session. In each replicate there were as many colonies under ‘informed’ as in the ‘naïve’ treatment. Replicates involving the same colonies were separated by more than one week to minimise memory of the previous situation, which is not expressed after 6 days [50].

### Data recording and analysis

Emigrations were observed until all new sites were discovered and we noted down the times at which intermediate and peripheral dishes were first entered by a worker. This allowed us to calculate an approximate crossing time for intermediate dishes (interval of time between the first entrance in the intermediate dish and the first entrance in the adjacent peripheral dish).

Additionally, all traffic to and from the new sites was recorded throughout emigration using a Webcam (Logitech ® QuickCam ® Communicate Deluxe with 1.3 Mp sensor) positioned above the nest entrance and connected to motion detection software Webcam Zone Trigger Version 2.300 Pro (Omega Unfold. Inc.), so that a picture was taken each time an ant entered or left the nest. Webcams were also present during the entire exploration period so that they would not constitute a novel landmark at the time of emigration. Analysis of pictures then allowed us to determine the emigration time for each colony (i.e. time interval between the start of emigration and the last transport of a brood item from the old nest to any new nest). Additionally, we determined for each new nest: i) the discovery time (interval from the time emigration was started to the time the new nest was first entered by a worker); ii) the assessment time (interval from the time the new nest was first entered by a worker to the time the first brood or adult was carried into the new nest); and iii) the transport time (interval from the time the first brood or adult was carried into the new nest to the time the last brood was carried into the new nest). Additionally, we counted the number of successful forward tandem runs (i.e. tandem runs were both leader and follower successfully entered the new nest). Monitoring all entrances and exits into and from the new nest sites allowed us to determine the population of workers in each site at each time; we could therefore determine an approximate quorum threshold for each nest (maximum population reached in the nest before the first brood or adult was carried).

In experiment 2 and 3, we took pictures of both new nest sites immediately after the end of emigration and (in experiment 3 only) 24 hours after the onset of emigration. A colony was deemed to have chosen a nest only if all brood items were in that nest; otherwise it was considered split. Additionally, we counted the total number of items (i.e. adults plus brood items) present in each nest using software ImageJ version 1.42q (National Institute of Health, USA). For each colony we then calculated a choosiness index (proportion of items observed in a given nest) and a cohesiveness index using the following formulas:
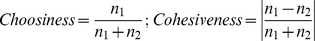
where *n_1_* and *n_2_* are the total number of items (i.e. adults and brood) in new nest sites 1 and 2 respectively; choosiness represented the degree of preference for nest site 1 whereas cohesiveness represented the degree of splitting, ranging from 0 (equal split of the colony between both nests) to 1 (choice of one single nest by the entire colony).

In experiment 3, colonies were monitored for 24 hours after the onset of emigration. For all colonies which had chosen a single nest after 24 hours, we defined a ‘Reunification time’ as the time interval between the start of emigration and the last item of brood carried into the chosen nest; this included both colonies which chose a single nest while emigrating and colonies which primarily split, then reunited after emigration. For data analysis, we only considered colonies which reunited in both treatments (n = 18).

### Statistical analyses

Statistical analyses were performed with software SPSS version 16.0 (SPSS Inc., Chicago IL), R version 2.10.1 and Minitab version 15.1.

Emigration-dynamic variables, quorum thresholds, number of forward tandem runs, choosiness and cohesiveness indexes were compared among treatments and nests using SPSS general linear mixed model procedure (GLMM) with fixed factors ‘Treatment’, ‘New nest site’ (if applicable) and their interaction, and random factors ‘Replicate’ and ‘Colony’. Normality and homoscedasticity of residuals were checked using Kolmogorov-Smirnov and Levene's tests, respectively. If residuals were not normally distributed, we applied either log- or power-transformation to the data. In cases where we could not identify any transformation allowing normalization of residuals we used non-parametric tests.

In experiment 1, the influence of the number of workers present in the familiar nest at the onset of emigration on quorum threshold and assessment time was investigated for informed colonies using SPSS linear regression and correlation procedures. For the regression, normality of residuals was checked using Kolmogorov-Smirnov tests.

In experiment 2 and 3, nest choice patterns were compared between treatments using two-tailed Fisher-Freeman-Halton's exact tests. Within treatments, nest preference was tested using exact binomial tests with a null hypothesis of random choice between both nests. Because there was a high splitting rate, nest preference was also tested using one-sample t-tests (normal samples) or one-sample Wilcoxon tests (non-normal samples) on choosiness indexes, with a null hypothesis of random choice between both nest, i.e. a hypothetic mean or median of 0.5.
